# Influence of oxidative stress on vascular calcification in the setting of coexisting chronic kidney disease and diabetes mellitus

**DOI:** 10.1038/s41598-020-76838-0

**Published:** 2020-11-26

**Authors:** Shuhei Watanabe, Hideki Fujii, Keiji Kono, Kentaro Watanabe, Shunsuke Goto, Shinichi Nishi

**Affiliations:** Division of Nephrology and Kidney Center, Kobe University Graduate School of Medicine, 7-5-2, Kusunoki-cho, Chuo-ku, Kobe, Hyogo 650-0017 Japan

**Keywords:** Kidney diseases, Nephrology

## Abstract

Vascular calcification (VC) is a common complication in patients with chronic kidney disease (CKD). Particularly, CKD patients with diabetes mellitus (DM) develop severe VC. Specific mechanisms of VC remain unclear; this study aimed to investigate them in the context of coexisting CKD and DM, mainly regarding oxidative stress. Sprague Dawley rats were randomly divided into six groups as follows: control rats (Control), 5/6 nephrectomized rats (CKD), streptozotocin-injected rats (DM), 5/6 nephrectomized and streptozotocin-injected rats (CKD + DM), CKD + DM rats treated with insulin (CKD + DM + INS), and CKD + DM rats treated with antioxidant apocynin (CKD + DM + APO). At 18 weeks old, the rats were sacrificed for analysis. Compared to the control, DM and CKD groups, calcification of aortas significantly increased in the CKD + DM group. Oxidative stress and osteoblast differentiation-related markers considerably increased in the CKD + DM group compared with the other groups. Moreover, apocynin considerably reduced oxidative stress, osteoblast differentiation-related markers, and aortic calcification despite high blood glucose levels. Our data indicate that coexisting CKD and DM hasten VC primarily through an increase in oxidative stress; anti-oxidative therapy may prevent the VC progression.

## Introduction

In patients with chronic kidney disease (CKD), cardiovascular disease (CVD) is a major cause of death^1–3^. The previous studies have reported that mortality resulting from CVD is higher in end-stage kidney disease (ESKD) patients than in the general population^4^. Vascular calcification (VC) is a type of cardiovascular lesions that is commonly observed in patients with CKD^5^. The prevalence and degree of VC increases with the CKD progression, and they are an independent predictor of CVD events and mortality^6^.

Needless to say, although CKD itself is a crucial risk factor for VC, several factors contribute to the pathogenesis of VC. Diabetes mellitus (DM) is particularly important among them. As DM is a major cause of ESKD, coexisting CKD and DM are commonly observed in clinical settings^7^. Moreover, patients with both CKD and DM are known to have higher incidences of CVD and all-cause mortality than those with either CKD or DM^8,9^. Considering these findings, it is important to evaluate the pathological mechanisms of VC in the setting of coexisting CKD and DM to improve their prognosis. However, to our knowledge, there is only a little information on this topic^10^, and the detailed mechanisms of VC in this specific context remain unclear. Particularly, there are few in vivo animal studies that involve both CKD and DM.

Oxidative stress, which is a risk factor for CKD and CVD, is known to rise in correlation with declining renal function^11–13^. The prognosis and occurrence of CVD events are associated with serum oxidative stress marker levels, which are elevated in patients with advanced-stage CKD^14,15^. Oxidative stress has been implicated in contributing to cardiac and kidney damage. Previously, we also reported that oxidative stress plays a crucial role in aggravating the organ damage, such as in the heart, kidneys, bones, and aortas of diabetic and CKD model rats^16–19^. Several experimental studies have indicated that various antioxidant approaches can attenuate the progression of organ damage in diabetic conditions^19–21^.

In this study, we developed a model rat with both CKD and DM and investigated the mechanisms of VC in the setting of coexisting of CKD and DM, particularly in the context of oxidative stress.

## Results

### Animal characteristics and biochemical measurements

The animal characteristics and biochemical data of the rats at 18 weeks old are shown in Table 1. Creatinine clearance (Ccr) was significantly lower in the CKD and CKD complicated by DM groups than in the control and DM groups and the treatment with INS or APO did not significantly change the kidney function. Blood glucose and HbA1c levels were higher in the DM, the CKD + DM and the CKD + DM + APO groups than in the control and CKD groups. These parameters also did not differ significantly between the CKD + DM and CKD + DM + APO groups. Serum phosphorus levels were significantly higher and serum calcium levels were significantly lower in the CKD and CKD complicated by DM groups than in the control and DM groups.

**Table 1:** Animal characteristics at 18 weeks old.

	Control (N = 6)	DM (N = 6)	CKD (N = 6)	CKD + DM (N = 6)	CKD + DM + INS (N = 6)	CKD + DM + APO (N = 6)
Body weight (g)	561 ± 9	432 ± 3^a^	403 ± 20^a^	286 ± 10^a,b,c^	408 ± 8^a,d^	298 ± 11^a,b,c,e^
SBP (mmHg)	120 ± 3	131 ± 0^a^	138 ± 1^a^	146 ± 4^a,b^	138 ± 2^a^	138 ± 2^a^
Alb (g/dL)	3.8 ± 0.1	3.1 ± 0.1^a^	3.4 ± 0.1^a^	2.7 ± 0.1^a,b,c^	3.1 ± 0.1^a,b^	2.7 ± 0.1^a,b,c,e^
BUN (mg/dL)	18.1 ± 0.5	21.6 ± 0.6	39.2 ± 2.9^a,b^	35.8 ± 1.0^a,b^	40.5 ± 2.0^a,b^	30.7 ± 2.5^a,b,c,e^
Ccr (mL/min)	4.2 ± 0.3	5.9 ± 0.4^a^	1.1 ± 0.1^a,b^	2.0 ± 0.2^a,b^	1.5 ± 0.1^a,b^	2.4 ± 0.3^a,b,c^
Blood glucose (mg/dL)	86 ± 3	360 ± 9^a^	83 ± 3^b^	407 ± 30^a,c^	108 ± 16^b,d^	341 ± 52^a,c,e^
HbA1c (%)	3.2 ± 0.1	9.2 ± 0.4^a^	3.3 ± 0.1^b^	9.5 ± 0.3^a,c^	3.8 ± 0.1^b,d^	9.3 ± 0.6^a,c,e^
Ca (mg/dL)	10.2 ± 0.1	9.4 ± 0.1	7.5 ± 0.3^a,b^	7.5 ± 0.4^a,b^	7.7 ± 0.3^a,b^	7.8 ± 0.2^a,b^
P (mg/dL)	8.3 ± 0.3	9.3 ± 0.1	16.3 ± 1.2^a,b^	15.6 ± 0.7^a,b^	15.5 ± 0.1^a,b^	13.6 ± 1.2^a,b^
iPTH (pg/mL)	974 ± 136	1357 ± 130	2937 ± 184^a,b^	1762 ± 121^a,c^	2914 ± 308^a,b,d^	1962 ± 125^a,c,e^

*DM* diabetes mellitus, *CKD* chronic kidney disease, *INS* insulin, *APO* apocynin, *SBP* systolic blood pressure, *Alb* albumin, *BUN* blood urea nitrogen, *Ccr* creatinine clearance, *HbA1c* hemoglobin A1c, *Ca* calcium, *P* phosphate, *iPTH* intact parathyroid hormone.

^a^Versus Control group, *P* < 0.05.

^b^Versus DM group, *P* < 0.05.

^c^Versus CKD group, *P* < 0.05.

^d^Versus CKD + DM, *P* < 0.05.

^e^Versus CKD + DM + INS, *P* < 0.05.

### Evaluation of aortic calcification

**Figure 1:**
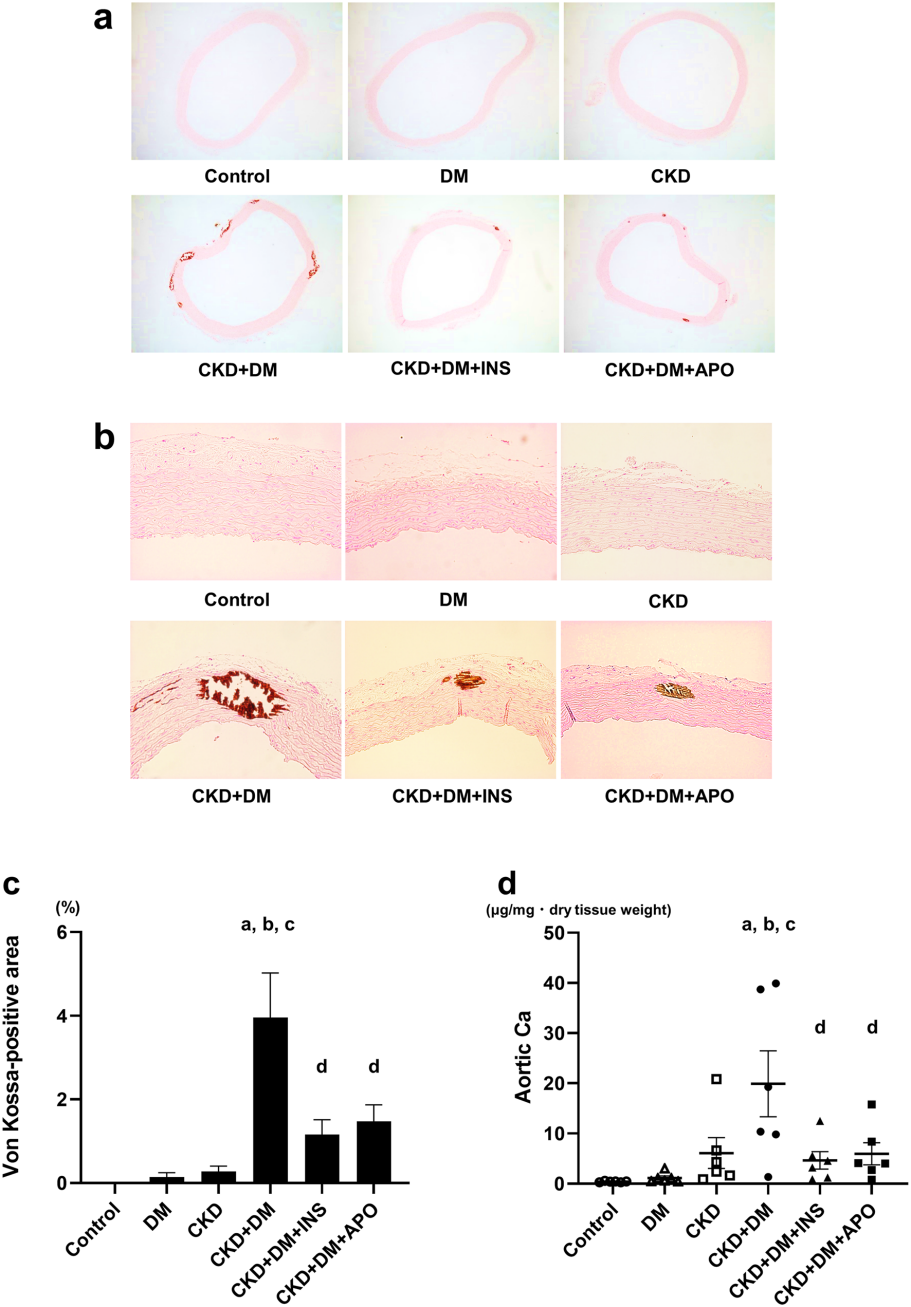
Evaluation of calcification of aortas. (**a**) Representative photomicrographs of the aorta with von Kossa stain (magnification, ×40). (**b**) Representavive photomicrographs of the aorta with von Kossa stain (magnification, ×200). (**c**) The percentage of von Kossa-positive mineralized area of aortas. (**d**) Calcium contents of aortas. DM, diabetes mellitus; CKD, chronic kidney disease; INS, insulin; APO, apocynin; Ca, calcium. ^a^Versus Control group, *P* < 0.05, ^b^versus DM group, *P* < 0.05, ^c^versus CKD group, *P* < 0.05, ^d^versus CKD + DM, *P* < 0.05.

We measured the percentage of the positive areas with von Kossa staining and the Ca contents of the aorta to assess the degree of aortic calcification in each group. Representative photomicrographs of the aorta stained with von Kossa staining are shown in Fig. 1a,b. Compared with the CKD and DM groups, the percentage of the positive areas with von Kossa staining was significantly higher in the CKD + DM group (Fig. 1c). However, compared with the CKD + DM group, the CKD + DM + INS and the CKD + DM + APO groups showed less aortic calcification. The presence of CKD and DM also increased the Ca contents of aortas, and the coexistence of these two conditions intensified the degree of calcification (Fig. 1d). Besides the results of the evaluation with von Kossa staining, the CKD + DM + APO group showed less aortic calcification than in the CKD + DM group, despite comparable blood glucose control.

### Evaluation of systemic and intraaortic oxidative stress

**Figure 2:**
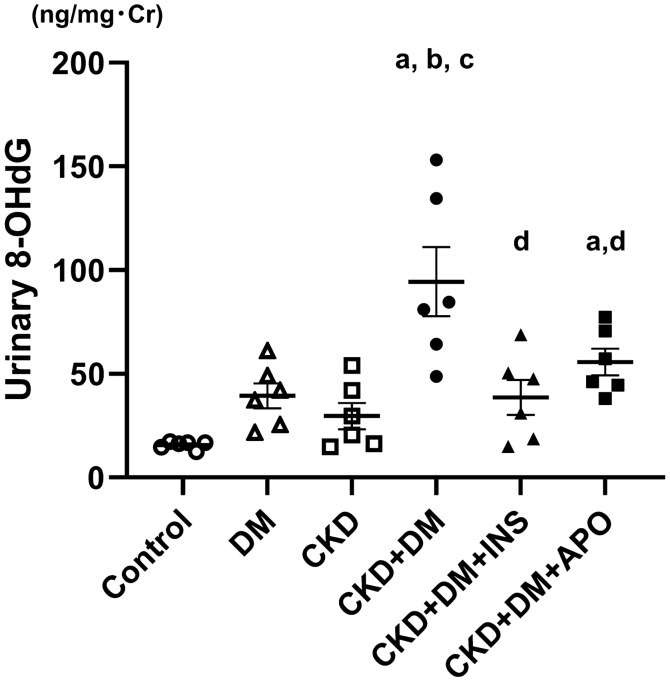
Evaluation of systemic oxidative stress. 8-OHdG, 8-hydroxy-2′-deoxyguanosine; DM, diabetes mellitus; CKD, chronic kidney disease; INS, insulin; APO, apocynin. ^a^versus Control group,* P* < 0.05, ^b^versus DM group,* P* < 0.05, ^c^versus CKD group, *P* < 0.05, ^d^versus CKD + DM, *P* < 0.05.

**Figure 3:**
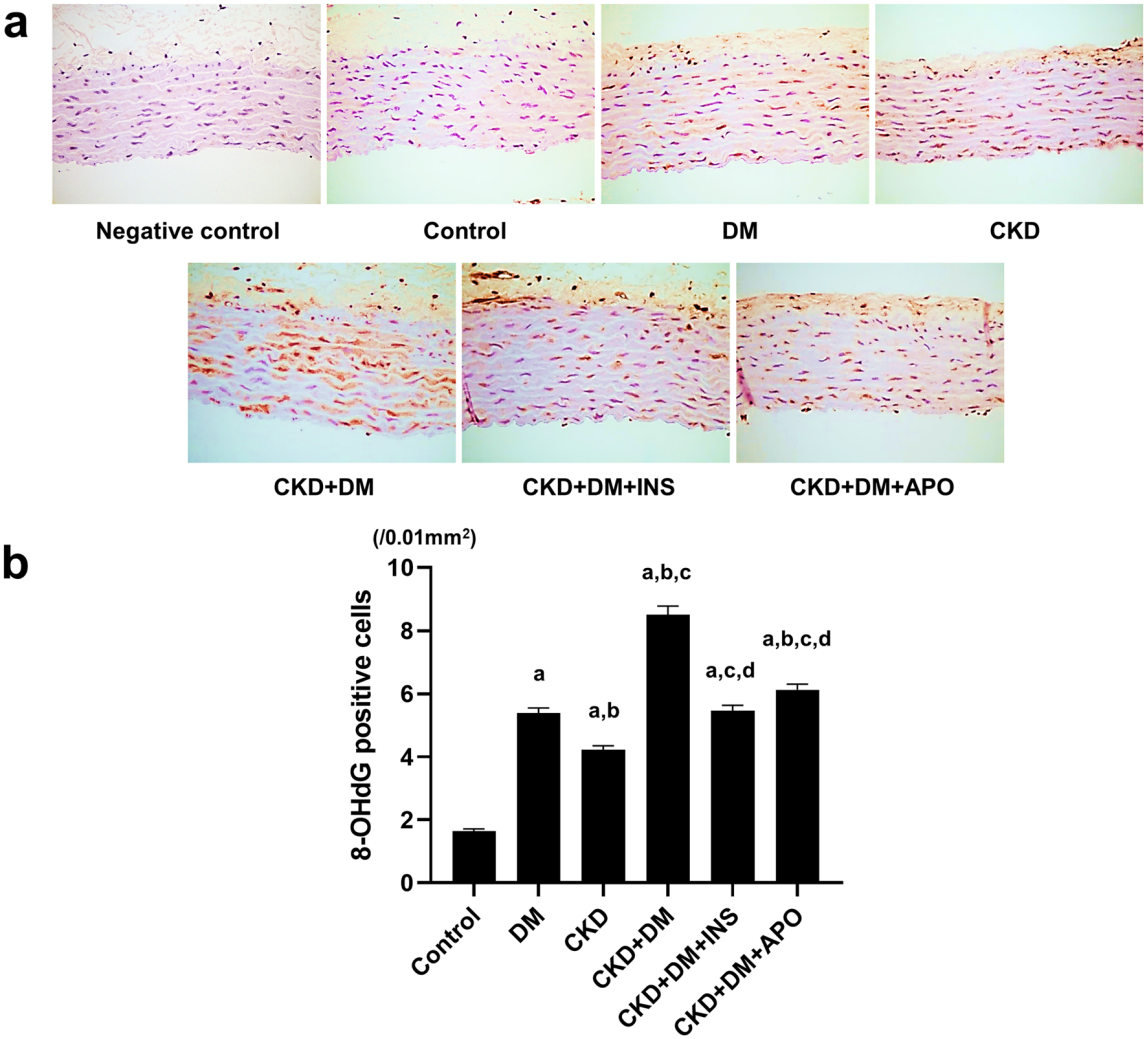
Evaluation of oxidative stress in aortas. (**a**) Representative photomicrographs of aortas with immunohistochemical staining for 8-OHdG (magnification, ×400). (**b**) The number of 8-OHdG positive cells in aortas. 8-OHdG, 8-hydroxy-2′-deoxyguanosine; DM, diabetes mellitus; CKD, chronic kidney disease; INS, insulin; APO, apocynin. ^a^versus Control group,* P* < 0.05, ^b^versus DM group,* P* < 0.05, ^c^versus CKD group, *P* < 0.05, ^d^versus CKD + DM, *P* < 0.05.

To evaluate systemic oxidative stress, we measured the urinary excretion of 8-hydroxydeoxyguanosine (8-OHdG). Urinary levels of 8-OHdG were corrected by urinary levels of creatinine for evaluation (Fig. 2). Urinary 8-OHdG excretion was higher in the DM, CKD, and CKD complicated by DM groups than in the control group (Control, 15.7 ± 0.8 ng/mg·Cr; DM, 39.4 ± 6.0 ng/mg·Cr; CKD, 29.6 ± 6.4 ng/mg·Cr), and the CKD + DM + INS and CKD + DM + APO groups revealed a marked decrease in oxidative stress compared with the CKD + DM group (CKD + DM, 94.4 ± 16.7 ng/mg·Cr; CKD + DM + INS, 38.5 ± 8.5 ng/mg·Cr; CKD + DM + APO, 55.7 ± 6.4 ng/mg·Cr). We also evaluated oxidative stress in the aortas with immunohistochemical methods. The 8-OHdG formation in aorta was significantly increased in the DM, CKD, and CKD complicated by DM groups compared with the control group (Fig. 3; Control, 1.6 ± 0.1/0.01 mm^2^; DM, 5.4 ± 0.2/0.01 mm^2^; CKD, 4.2 ± 0.1/0.01 mm^2^; CKD + DM, 8.5 ± 0.3 /0.01 mm^2^; CKD + DM + INS, 5.5 ± 0.2 /0.01 mm^2^; CKD + DM + APO, 6.1 ± 0.2 /0.01 mm^2^). The treatment with APO as well as INS reduced intraaortic oxidative stress compared with the CKD + DM group.

**Figure 4:**
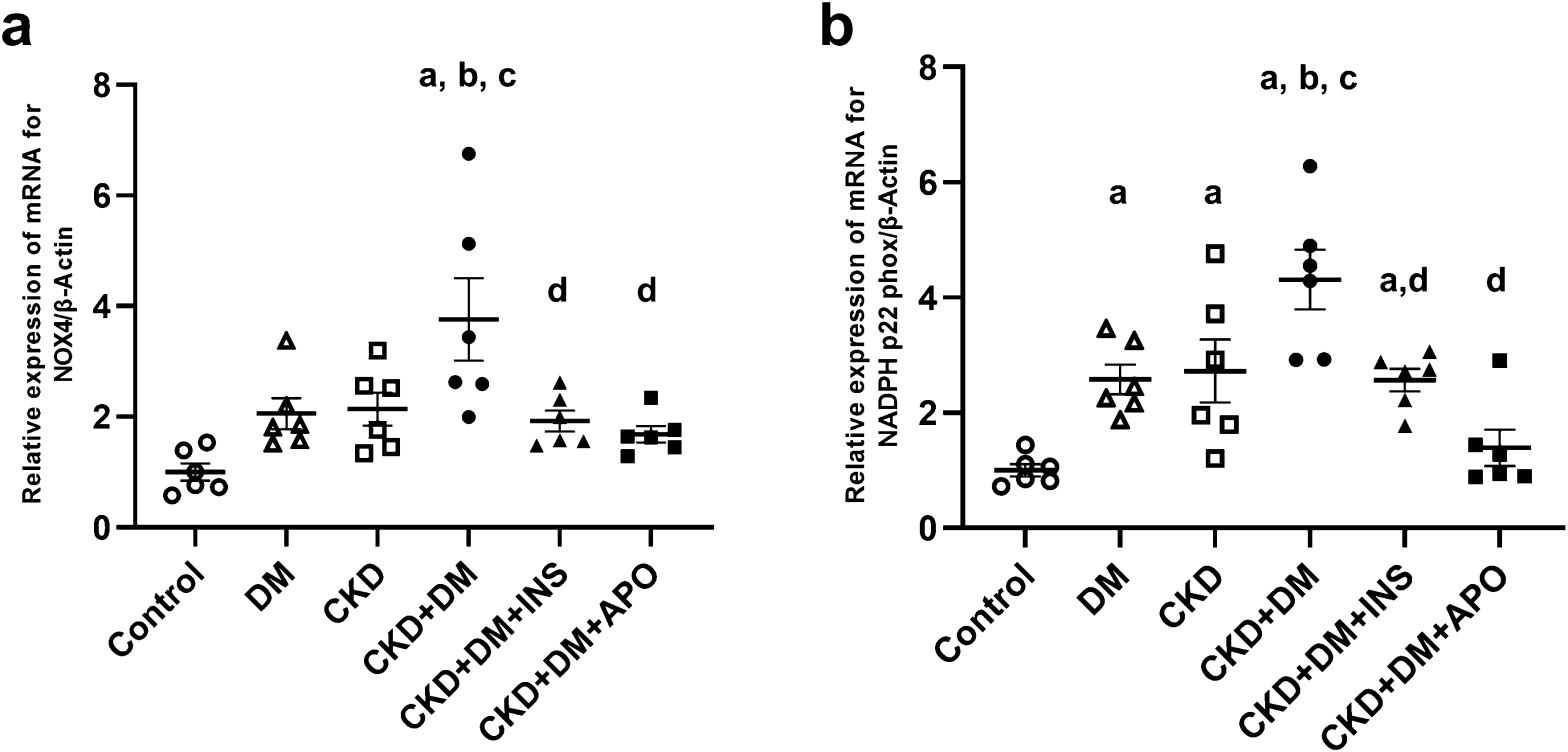
mRNA expression of NADPH oxidase. (**a**) NOX4. (**b**) NADPH p22 phox. NOX4, nicotinamide adenine dinucleotide phosphate oxidase 4; NADPH, nicotinamide adenine dinucleotide phosphate oxidase; DM, diabetes mellitus; CKD, chronic kidney disease; INS, insulin; APO, apocynin. ^a^versus Control group,* P* < 0.05, ^b^versus DM group,* P* < 0.05, ^c^versus CKD group, *P* < 0.05, ^d^versus CKD + DM, *P* < 0.05.

Moreover, the mRNA expression of nicotinamide adenine dinucleotide phosphate (NADPH) oxidase was remarkably increased in the CKD + DM groups compared with the control group (Fig. 4). The expressions of oxidative stress-related markers were also significantly decreased in the CKD + DM + INS and CKD + DM + APO groups compared with the CKD + DM group.

### Evaluation of osteoblast differentiation-related markers

**Figure 5:**
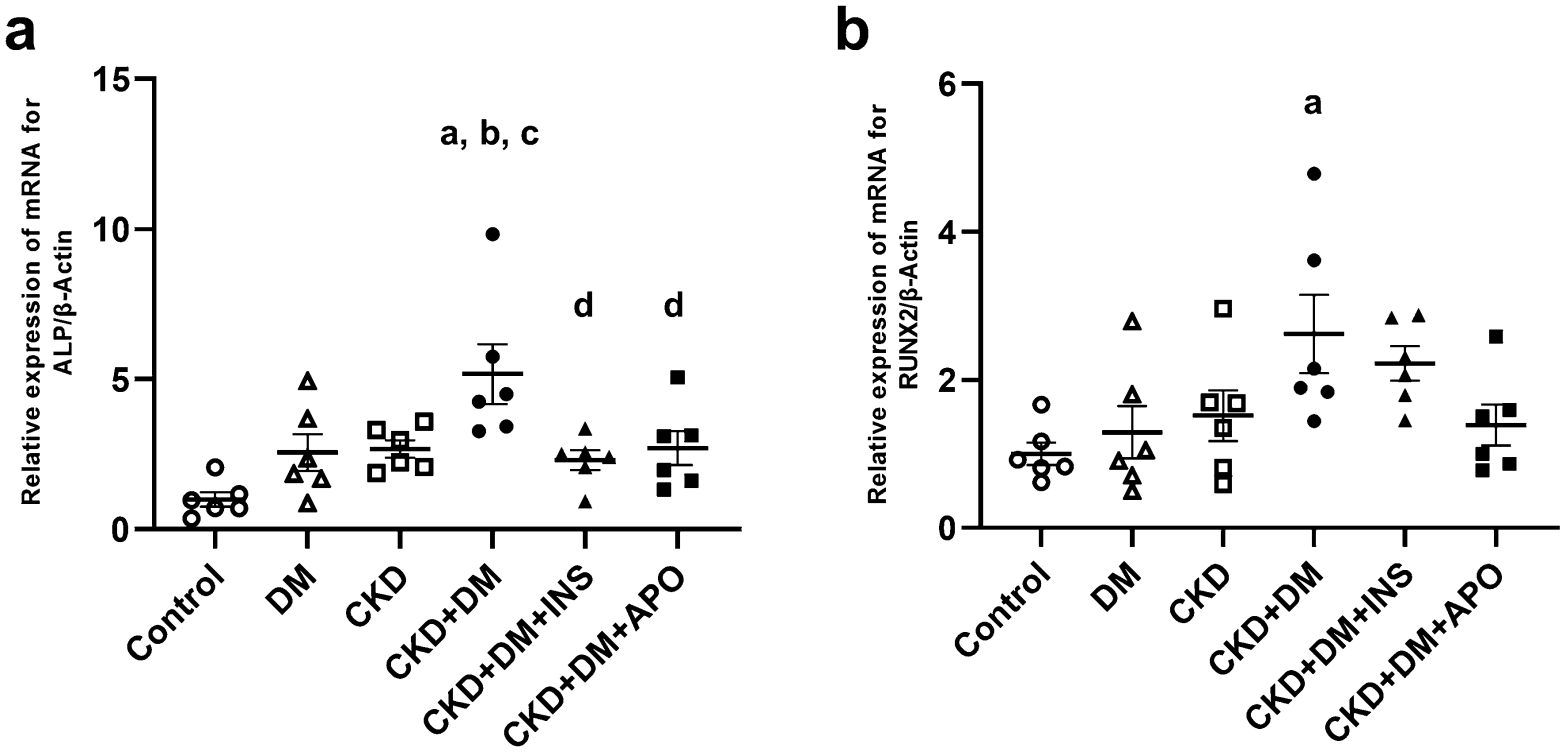
mRNA expression of osteoblast-differentiation markers. (**a**) ALP. (**b**) RUNX2. ALP, alkaline phosphatase; RUNX2, Runt-related transcription factor 2; DM, diabetes mellitus; CKD, chronic kidney disease; INS, insulin; APO, apocynin. ^a^versus Control group,* P* < 0.05, ^b^versus DM group,* P* < 0.05, ^c^versus CKD group, *P* < 0.05, ^d^versus CKD + DM, *P* < 0.05.

**Figure 6:**
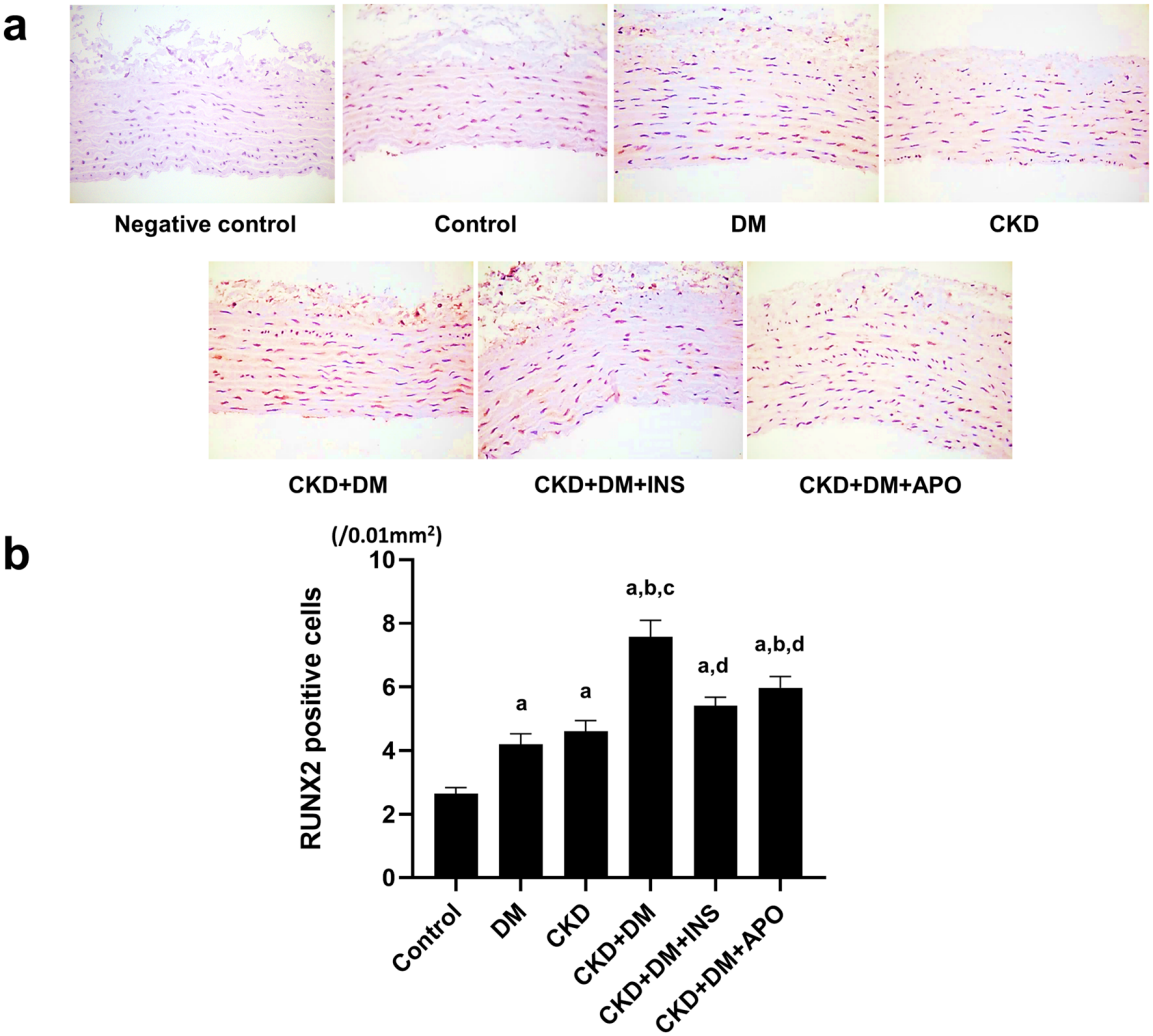
Evaluation of RUNX2 expression with immunohistochemical staining. (**a**) Representative photomicrographs of aortas with immunohistochemical staining for RUNX2 (magnification, ×400). (**b**) The number of RUNX2 positive cells in aortas. RUNX2, Runt-related transcription factor 2; DM, diabetes mellitus; CKD, chronic kidney disease; INS, insulin; APO, apocynin. ^a^versus Control group,* P* < 0.05, ^b^versus DM group,* P* < 0.05, ^c^versus CKD group, *P* < 0.05, ^d^versus CKD + DM, *P* < 0.05.

To evaluate the known osteogenic markers related to VC, we evaluated mRNA expression of alkaline phosphatase (ALP) and Runt-related transcription factor 2 (RUNX2) in the aortas. The mRNA expressions of ALP and RUNX2 were higher in the DM, CKD, and three CKD + DM groups than in the control group (Fig. 5). The immunohistochemical staining of RUNX2 also showed the number of RUNX2 positive cells was significantly higher in the CKD + DM group than in the control, CKD, and DM groups (Fig. 6). Similarly, these expression of osteoblast differentiation-related markers were significantly decreased in the CKD + DM + APO group, as well as in the CKD + DM + INS group, compared with the CKD + DM group.

### Relationship between oxidative stress, osteoblast differentiation-related markers, and calcification

**Figure 7:**
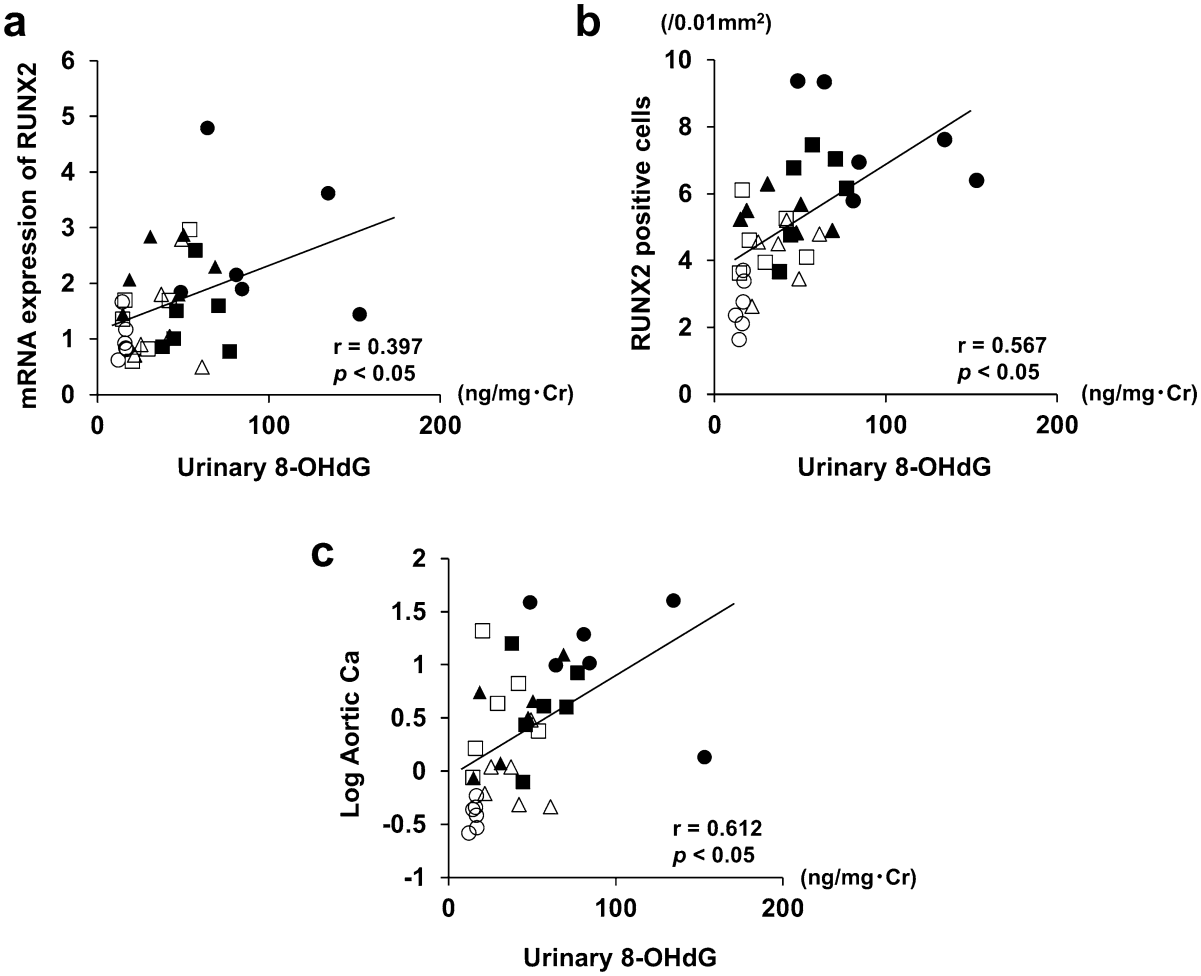
Correlation of oxidative stress with RUNX2 and calcification. (**a**) Relationship between urinary 8-OHdG excretion and mRNA expression of RUNX2 in aortas. Urinary 8-OHdG excretion was significantly associated with mRNA expression of RUNX2 (r = 0.397, *P* < 0.05). (**b**) Relationship between urinary 8-OHdG excretion and the number of RUNX2 positive cells in aortas. Urinary 8-OHdG excretion was significantly associated with the number of RUNX2 positive cells in aortas (r = 0.567, *P* < 0.05). (**c**) Relationship between urinary 8-OHdG excretion and calcium contents of aortas. Urinary 8-OHdG excretion was significantly associated with calcium contents of aortas (r = 0.612, *P* < 0.05). Symbols denote the same group in each figure (Control group, open circle; DM group, open triangle; CKD group, open square; CKD + DM group, closed circle; CKD + DM + INS group, closed triangle; CKD + DM + APO group, closed square). 8-OHdG, 8-hydroxy-2′-deoxyguanosine; RUNX2, Runt-related transcription factor 2; Ca, calcium.

We analyzed the correlation of systemic oxidative stress with the RUNX2 expression in the aortas and calcium content of the aorta. Urinary 8-OHdG excretion was significantly correlated with the mRNA expression of RUNX2 and the number of RUNX2-positive cells in the aortas (Fig. 7a: mRNA expression of RUNX2, r = 0.397, *p* < 0.05; Fig. 7b: the number of RUNX2-positive cells, r = 0.567, *p* < 0.05). Moreover, urinary 8-OHdG excretion was also significantly correlated with the calcium content of the aortas (Fig. 7c: r = 0.612, *p* < 0.05).

## Discussion

In this study, we demonstrated that (1) von Kossa-positive mineralized area and calcium content of aortas were higher in the CKD + DM group than in the control, CKD, and DM groups; (2) the expressions of osteoblast differentiation-related markers and oxidative stress-related markers were increased in the CKD + DM group compared with the control, CKD, and DM groups; (3) the degree of VC and oxidative stress was significantly lower in the CKD + DM + APO group than in the CKD + DM group; and (4) oxidative stress-related markers were significantly and positively correlated with osteoblast differentiation-related markers.

VC is a major risk factor for CVD^22^, and exploring strategies for the prevention of VC progression is very important. Although DM and CKD are known to play key roles in VC progression, few studies elucidated how their coexistence impacts its progression. Our previous observational study showed that the presence of DM was significantly associated with the degree of aortic calcification in patients with stages 4 and 5 CKD^23^. After adjustment for several confounding factors, DM was one of the most important risk factors for VC in these advanced-stage CKD patients. In patients undergoing hemodialysis, a significantly positive association was observed between hemoglobin A1c levels and the degree of VC^24^. Although serum phosphate level is recognized as a risk factor for VC, a previous study reported that mineral bone disorder was not significantly associated with VC in diabetic patients undergoing hemodialysis^25^. Considering these results, DM is seen to be a crucial condition for VC progression even in CKD.

The mechanisms underlying VC progression in CKD and DM are complicated, and several factors contribute to its pathogenesis. There are many reports about the mechanisms of VC progression in the field of CKD and DM; however, few studies focused on the VC progression in CKD complicated by DM. Many factors such as hyperlipidemia, hypertension, DM, aging, and mineral bone disorder, are considered to be related to the pathophysiological mechanisms of VC^26,27^. Among them, we focused on oxidative stress in this study. Many studies have shown that oxidative stress may contribute to the pathogenesis of VC progression in a rat CKD and DM model^28,29^. Our several previous studies also proved that an increase in oxidative stress was associated with the progression of kidney and cardiovascular injury in not only CKD but also DM^16–18,30^. In this study, both the CKD and DM groups manifested increased oxidative stress and rats with CKD complicated by DM exhibited increased oxidative stress compared with them.

NADPH oxidases are OHdG-generating enzymes and have been implicated in the pathophysiology of various type of organ damage^31^. Studies on the CV system have reported that NADPH oxidases promote endothelial dysfunction, inflammation, cardiac hypertrophy, fibrosis, apoptosis, and remodeling in vessel walls and the heart^32,33^. Our previous experimental study using diabetic model rats demonstrated that the mRNA expression of NADPH oxidase considerably increased in aortic tissue compared with the control rats and glycemic control with insulin suppressed the increase^16^. Moreover, another study using CKD model rats showed that the mRNA expression of NADPH oxidase in aortic tissue and urinary excretion of 8-OHdG increased, and urinary excretion of 8-OHdG was significantly correlated with the degree of VC^28^. We also found that the mRNA expression of NADPH oxidase significantly increased in aortic tissue in the CKD + DM group compared with the CKD and DM groups. Urinary excretion of 8-OHdG and the expression of 8-OHdG in the aortic tissue also revealed a remarkable increase in the CKD + DM group. To our knowledge, the present study is the first to examine oxidative stress in the context of coexisting CKD and DM. In clinical study, it has been reported that hemodialysis patients with DM had significantly higher oxidative albumin levels compared with those without DM^34^. Another study demonstrated that increased NADPH oxidase-mediated superoxide production was associated with enhanced coronary artery calcification in the general population^35^. Taken together, increased oxidative stress enhanced by both CKD and DM can play a key role in the VC progression.

Regarding the pathogenesis of VC progression, the effect of oxidative stress on vascular smooth muscle cells (VSMCs) is considered to be crucial^36–38^. The formation and accumulation of advanced glycation end products (AGEs) are accelerated in the context of CKD and DM^29,39^. AGE interacts with the receptor for AGE, leading to vascular injury by evoking oxidative stress^40^. An in vitro study showed that AGE induced calcium deposition in VSMCs through increases in the mRNA expression of NADPH oxidase and 8-OHdG concentration in the medium^41^. Moreover, AGE increased the mRNA expression of osteoblast differentiation-related markers as well as oxidative stress-related markers in VSMCs. Although we could not evaluate AGE, the results of our study also showed that urinary 8-OHdG excretion was significantly correlated with the RUNX2 expression and the calcium content of the aorta. Oxidative stress has been reported to induce the expression of bone markers and VSMC calcification through the activation of the AKT signaling pathway and downregulation of VSMC markers^36^. We confirmed that the expressions of ALP and RUNX2 in the aorta were highest in the CKD + DM group. From these results, it was gathered that oxidative stress induced differentiation of VSMC to osteoblast, and leading to VC progression.

From the clinical setting perspective, it has been speculated that the prevention of VC progression can reduce CVD events and mortality. Therefore, a treatment that halts VC progression would be of great interest to many clinicians. The suppression of oxidative stress has been verified as one such treatment. A randomized controlled trial for patients undergoing maintenance hemodialysis demonstrated that the antioxidant acetylcysteine significantly reduced CVD events compared with the placebo^42^. In this study, we used apocynin to examine the effect of antioxidative therapy on VC. Treatment with apocynin decreased aortic calcification and osteoblast differentiation-related markers through the suppression of oxidative stress in the context of coexisting CKD and DM. Since the streptozotocin-induced diabetes is characterized by insulin deficiency, these results could not apply to all diabetic patients. However, impairment of insulin secretion is a more important pathophysiological condition in type 1 diabetic and Asian type 2 diabetic patients. Many patients with type 2 diabetes in Asia often show non-obesity and impairment of insulin secretion. Therefore, we think the results of the present study could be meaningful particularly for such diabetic conditions.

Our data suggest that antioxidative therapy could be useful for the prevention of VC progression in CKD and DM. To ascertain clinical efficacy, further clinical study is needed.

## Methods

### Animals and experimental protocol

Six-week-old male Sprague Dawley (SD) rats were obtained from CLEA Japan Inc. (Tokyo, Japan). The rats were housed with ad libitum access to food and water and in light- and temperature-controlled environments. At 7 weeks old, the rats were randomly divided into four groups as follows; sham-operated SD (control, n = 6), sham-operated and induced-DM SD (DM, n = 6), induced-CKD SD (CKD, n = 6), and induced-CKD and induced-DM SD (CKD complicated by DM, n = 18).

At 7 weeks age, the rats in the CKD and CKD complicated by DM groups were briefly anesthetized with sodium pentobarbital and underwent two-thirds nephrectomies of the right kidneys. One week later, the remaining left kidneys were resected. The rats in the control and DM group underwent sham operations at the same time. At 9 weeks old, rats in the DM and CKD complicated by DM groups were injected, under anesthesia, with streptozotosin (STZ; 55 mg/kg body weight, Sigma Chemical Co., St louis, MO, USA) dissolved in 0.1 mol/l sodium citrate buffer, pH 4.5, via tail veins. The rats in the control and CKD groups were injected with an equal amount of citrate buffer alone. One week later, tail vein blood glucose was determined using a Glutest Ace (Sanwa Kagaku Kenkyusho, Nagoya, Japan). Rats treated with STZ were considered to have DM when blood glucose levels were higher than 300 mg/dl. At 11 weeks old, the rats in the CKD complicated by DM group were further divided into three groups as follows: the rats with no treatment (CKD + DM, n = 6), the rats treated with insulin (CKD + DM + INS, n = 6), and the rats treated with an antioxidant agent, apocynin (CKD + DM + APO, n = 6). Lastly, the rats were divided into six groups as described above.

At 11 weeks old, the rats in the CKD + DM + INS group were treated with insulin implants (LinShin Canada Inc., Scarborough, Ont., Canada), which release a controlled amount of insulin. The rats in the CKD + DM + APO group were given drinking water containing three mmol/l apocynin (Tokyo Chemical Industry, Co., Ltd., Tokyo, Japan) until the end of the study period. Apocynin has been used in experimental studies as an antioxidant agent and inhibits NADPH oxidases and scavenges reactive oxygen species (ROS)^43^.

The rats in all groups were fed a high phosphate diet containing 1.0% calcium and 2.0% phosphate. Before the rats were sacrificed, 24 h urine samples were collected from each rat using a metabolic cage. At 18 weeks old, these rats were sacrificed under sodium pentobarbital anesthesia. Blood samples were collected from the left ventricles for serum biochemical analysis, and the aortas were removed for RNA extraction and histomorphological analysis.

This study was conducted in strict accordance with the recommendations in the Guide for the Care and Use of Laboratory Animals of the National Institutes of Health. The Institutional Animal Care and Use Committee at Kobe University Graduate School of Medicine approved the protocol (Permit Number: P150507). All surgeries were conducted under sodium pentobarbital anesthesia, and every effort was made to minimize suffering, following the ARRIVE guidelines for reporting experiments involving animals^44^.

### Blood and urine analysis

After 3000 rpm centrifugation for 10 min, the serum and urine samples were stored at – 80 °C until analysis. Serum creatinine (Cr) and urea nitrogen levels were measured using Fuji Dri-Chem 3500 (Fujifilm Japan, Tokyo, Japan). Urine Cr and serum calcium and phosphate levels were measured at SRL Inc (Tokyo, Japan). HbA1c levels were determined with a DCA 2000 analyzer (Bayel Medical, Tokyo, Japan), and glucose levels were determined using a Glutest Ace with venous blood samples obtained from tails. As a sensitive marker of oxidative stress, the urinary excretion of 8-OHdG was detected using an ELISA kit (Japan Institute for Control of Aging, Shizuoka, Japan). The plasma parathyroid hormone levels were measured using an ELISA kit (Immunotopics, San Clemente, CA, USA).

### Blood pressure measurements

Systolic blood pressure was measured by tail-cuff plethysmography (Model MK-2000; Muromachi Kikai CO., Ltd., Tokyo, Japan). To reduce the possibility of stress artifacts, the measurements were taken after a sufficient acclimatization period. Systolic blood pressure was determined by multiple readings for each rat at baseline and the end of the study period.

### Evaluation of vascular calcification

To quantify calcium content, the lower parts of the abdominal aortas were removed. The aortas were freeze-dried, weighed, and then incubated in 0.6 N HCl for 48 h. The samples were centrifuged and the supernatant were analyzed for calcium contents using the Calcium-E test (Wako, Osaka, Japan). Aortic calcium content was corrected for the dry tissue weight. The thoracic aortas were removed and fixed in 10% formaldehyde for histopathology. These sections were dehydrated with 70% ethanol at room temperature and embedded in paraffin blocks, which were deparaffinized and processed for von Kossa staining. The percentage of the positive areas of von Kossa staining in the aortic sections were calculated in 30 randomly selected microscopic fields using Lumina Vision image analysis software version 3.7.4.2 (Mitani Co., Tokyo, Japan). All evaluations were conducted in a blinded manner.

### Immunohistochemical analyses

The thoracic aortas in paraffin blocks were deparaffinized and processed for immunohistochemical staining. The formation of 8-OHdG was evaluated with anti-8-OHdG monoclonal antibodies raised in rats (Japan Institute for Control of Aging, Shizuoka, Japan). The expression of RUNX2, which is a marker of osteoblast differentiation, was evaluated with anti-RUNX2 antibody (Abcam, Cambridge, United Kingdom). Sectioning and staining were conducted according to standard procedure of Morpho Technology (Sapporo, Japan). To give 8-OHdG-positive and RUNX2-positive cell scores, the 8-OHdG-positive and RUNX2-positive cells in the aortic tissue were counted in 30 random microscopic fields. The results of the positive cells were expressed as the number of positive cells per 0.01 mm^2^ of the examined area.

### RNA extraction and real-time polymerase chain reaction

The upper parts of the abdominal aortas were removed and snap-frozen using liquid nitrogen. Then, these samples were then stored at -80℃ until evaluation with real-time polymerase chain reaction (PCR). Using an ISOGEN kit (Wako Pure Chemicals Industries, Ltd., Osaka, Japan), total RNA was extracted from the samples of aortas according to the manufacturer’s instructions. Total RNA from the rat aortas was used as the template for cDNA synthesis with the ReveTra ACE qPCR RT kit (TOYOBO Co., Ltd., Osaka, Japan) using an oligo-dT primer as per the manufacturer’s recommended protocol. The synthesized cDNA was stored at -80℃ until analysis with quantitative PCR. mRNA expression was examined with real-time PCR using the Light Cycler 350 s Real-Time PCR System (Roche Diagnostics, Mannheim, Germany) with the SYBR Green Assay with Thunderbird SYBR qPCR Mix (TOYOBO Co., Ltd., Osaka, Japan) following the manufacturer’s protocol. The analysis was performed with the second derivative maximum method of the LightCycler software (version. 4.0; Roche). The relative amount of the sample mRNA was normalized to the β-actin mRNA. For PCR analysis, we used the following primers; rat NADPH oxidase 4 (NOX4; 5′-GAACCCAAGTTCCAAGCTCA-3′, 5′-GCACAAAGGTCCAGAAATCC-3′), rat NADPH p22 phox (5′-GGTGAGCAGTGGACTCCCATT-3′, 5′-TGGTAGGTGGCTGCTTGATG-3′), rat Runt-related transcription factor 2 (RUNX2; 5′-AAGTGCGGTGCAAACTTTCT-3′, 5′-TCTCGGTGGCTGGTAGTG-3′), rat Alkaline Phosphatase (ALP; 5′-TCCGTGGGTCGGATTCCT-3′, 5′-GCCGGCCCAAGAGAGAAA-3′), rat β-Actin (5′-TGACAGGATGCAGAAGGAGA-3′, 5′-TAGAGCCACCAATCCACACA-3′).

### Statistical analysis

All data are expressed as mean ± SEM. One-way analysis of variance, followed by the Tukey–Kramer test, was used for comparison of each group. Pearson’s correlation coefficient or Spearman rank correlation coefficient was used to analyze relationships between variables as appropriate. A *p* value of < 0.05 was considered statistically significant. All statistical analyses were conducted using IBM SPSS Statistics version 23.0 (Chicago, IL, USA).

## Data Availability

The datasets generated during and/or analysed during the current study are available from the corresponding author on reasonable request.
